# New Lipase for Biodiesel Production: Partial Purification and Characterization of LipSB 25-4

**DOI:** 10.1155/2014/289749

**Published:** 2014-03-10

**Authors:** Aysel Ugur, Nurdan Sarac, Rukiye Boran, Berk Ayaz, Ozgur Ceylan, Gulten Okmen

**Affiliations:** ^1^Section of Medical Microbiology, Department of Basic Sciences, Faculty of Dentistry, Gazi University, TR06510 Ankara, Turkey; ^2^Department of Biology, Faculty of Sciences, Mugla Sıtkı Kocman University, TR48000 Mugla, Turkey; ^3^Medical Laboratory Program, Vocational School of Health Service, Aksaray University, TR68000 Aksaray, Turkey; ^4^Apiculture Program, Vocational School of Ula Ali Kocman, Mugla Sıtkı Koçman University, TR48640 Mugla, Turkey

## Abstract

The lipolytic activities of 300 *Streptomyces* isolates were determined in Tributyrin and Rhodamine-B Agar. Lipase activities were also measured with *p*-nitrophenyl palmitate (*p*-NPP) as a substrate. The strain of *Streptomyces bambergiensis* OC 25-4 used in this study was selected among 300 strains of Streptomyces from MUCC as the best lipase producer. The incubation conditions were optimized and the inoculum amount, incubation period, effect of carbon and nitrogen sources, and rates of MgSO_4_ and CaCO_3_ were investigated. LipSB 25-4 (the lipase produced by *S. bambergiensis* OC 25-4 strain) was partially purified with ammonium sulphate precipitation, dialysis, and gel filtration chromatography 2.73-fold and with 92.12 U/mg specific activity. The optimal pH and temperature for LipSB 25-4 were determined as 8.0 and 50°C, respectively. The lipase has high stability in all pH and temperature values used in this study. While LipSB 25-4 was slightly activated in the presence of *β*-mercaptoethanol, it was slightly reduced by PMSF. The enzyme conserved approximately 75% of its activity at the end of 60 h, in the presence of methanol and ethanol. Since LipSB 25-4 displays high activity in the thermophilic conditions and stability in the presence of organic solvents, this lipase can catalyse the biodiesel production from olive oil by the transesterification reactions.

## 1. Introduction

The increasing severity of the global energy crisis, shortage of fossil fuels, increase in the crude oil prices, and an increasing number of environmental problems and environmental concerns to reduce pollution have resulted in the rapid growth of research into alternative energy sources, as well as the use of such sources [1, 2]. Biodiesel which is derived from triglycerides by transesterification with methanol is receiving increasing attention as an alternative, nontoxic, biodegradable, and renewable source of fuel and energy with significantly lower exhaust emissions of particulate matter and green-house gases [3–5] and for its ability to replace fossil fuels. Chemically, biodiesel is produced by transforming triglycerides into fatty acid alkyl esters in the presence of alcohol, such as methanol or ethanol, and an acid or alkali catalyst, generating glycerol as a by-product [6]. Enzymes represent an environmentally friendly alternative to chemical catalysts [7].

Utilization of lipase as a catalyst for biodiesel fuel production has great potential compared with chemical methods [7, 8]. Recently, lipase-catalyzed methanolysis method has become more attractive than the traditional chemical means in biodiesel production, because the conventional biodiesel production, which involves the use of chemical catalyst is carried out at relatively high temperatures closer to the boiling point of the alcohol and produces many unwanted by-products like soap. Separation of biodiesel from these by-products and glycerol is difficult, which renders this method costly and complicated [9, 10]. For these reasons, researchers have attempted to locate and identify other more suitable lipase enzymes. The most suitable enzyme in this regard must possess not only the ability to produce biodiesel efficiently using oil [11] but also the ability to utilize all mono, di, and triglycerides as well as high yield of free fatty acids, low product inhibition, high activity and yield in nonaqueous media, low reaction time, temperature and alcohol stability, reusability of immobilized enzyme, and so forth [1].

Lipases (triacylglycerol acylhydrolases, EC 3.1.1.3), acting only on ester-water interface, can hydrolyze long-chain triglycerides to diacylglycerol and carboxylate, as well as the reverse reaction, synthesis of esters from fatty acids and glycerol [12]. The main industrial application of lipases is in the hydrolysis of fats and oils [13], although their use in the transesterification of oils for the synthesis of biodiesel is increasing [14, 15]. Commercially useful lipases are usually obtained from microorganisms that produce a wide variety of extracellular lipases [16].

The genus* Streptomyces* consists of sporulating Gram-positive soil bacteria with a mycelial growth habit and a life cycle with complex morphological and physiological differentiation [17].* Streptomyces* species are among the best-studied and best-characterized bacteria due to their significant role in medical science, ecology, and the biotech industry [18].* Streptomyces* strains were recognized through their high exogenous lipolytic activity; lipases of this genus were not studied as intensively as were those from some other bacteria [19].

This paper deals with the screening of 300* Streptomyces* isolates obtained from Mugla University Culture Collection (MUCC) for lipase production, and then optimization of growth conditions for maximum enzyme production, partial purification, characterization, and application of the enzyme from* Streptomyces bambergiensis* OC 25-4 in biodiesel production. This is the first report that lipase from* S. bambergiensis* could be used in biodiesel production.

## 2. Materials and Methods

### 2.1. Screening of Lipolytic* Streptomyces* Isolates

Qualitatively, the lipolytic activity of the* Streptomyces* isolates which was obtained from MUCC was screened using Tributyrin Agar (TA) plates [composition, g/L: peptone 5.0, beef extract 3.0, tributyrin 15 mL (v/v), agar-agar 15.0, pH 7.0 ± 0.2]. The isolates were streaked on TA plates and incubated at 30°C for 168 h. Lipolytic activity was observed as a zone of hydrolysis around the bacterial colonies.

Lipolytic activity was further confirmed by streaking the positive isolates on Rhodamine-B Agar plates. The medium contains 0.8% of nutrient broth (NB), 0.4% NaCl, 2.5% olive oil, 1% of agar-agar, and 1‰ Rhodamine B. The isolates were incubated for 168 h at 30°C in this medium, and the lipolytic activity was screened under UV light which appeared as an orange fluorescent zone around the bacterial colonies [20].

### 2.2. Enzyme Production

The isolates, which have lipolytic activities, were inoculated on ISP2 medium and incubated at 30°C for 7 days. After the incubation, 0.01% (v/v) Tween 80 solution was used to harvest the spores [21]. For this purpose, Tween 80 solution was added onto the surface of sporulated isolate under aseptic conditions. After 10 minutes, the spores crossed the solution, and the solution was taken in a sterile tube.

Flasks of the ISP2 broth were inoculated with 2% spore solution and incubated on a rotary shaker at 130 rpm, 30°C for 7 days. After the incubation, the cells were filtrated with Whatman filter paper number 42, and the supernatant was used as the source of extracellular lipase.

### 2.3. Lipase Assay

Lipase assay was performed according to [22], with some modifications [23]. A quantity of 30 mg of* p*-NPP was dissolved in 10 mL isopropanol, emulsified with 0.1 g Arabic gum, and to this was added 2 mL Triton X-100. The reaction mixture was prepared by adding this emulsion to 90 mL Tris-HCl buffer (50 mM, pH 8). Then, 1 mL of enzyme solution was added to 9 mL of substrate solution, the mixture was incubated at 30°C for 30 min, and absorbance was measured at *λ* = 410 nm in a spectrophotometer.

### 2.4. Optimization of Growth Conditions

The optimum inoculum amount of spore solution of the maximum lipase producer isolate was determined. The carbon and nitrogen sources, rates of MgSO_4_ and CaCO_3_, were optimized. Starch, sucrose, olive oil, and olive oil+ glucose were added to the growth medium as a carbon source. As a nitrogen source, ammonium sulphate, sodium nitrate, sodium nitrate+ yeast extract, meat peptone, and soybean peptone were added to the growth medium.

The optimum time course of lipase production of this isolate was studied for 180 h. The spore solution was added 2% (v/v) to 50 mL ISP2 broth medium in an Erlenmeyer flask (250 mL) and incubated at 130 rpm on a rotary shaker, at 30°C, for 180 h. The culture was taken periodically and the lipase activity in the culture supernatant were determined.

### 2.5. Enzyme Purification

The enzyme from the supernatant was precipitated with ammonium sulfate at 90% saturation and pH 5.0; the precipitate was dissolved in 50 mM Tris-HCl buffer (pH 8) and then dialyzed in 50 mM Tris-HCl buffer (pH 8). The enzyme preparation was loaded on a Sephacryl S-100 HR column preequilibrated with 50 mM Tris-HCl buffer (pH 8). The sample was eluted with 50 mM Tris-HCl buffer (pH 8). Four-milliliter fractions were collected from the column and assayed for lipase activity. In addition, that enzyme was concentrated with an ultrafiltration membrane. In this study, the protein concentrations were determined according to the method of Bradford by using bovine serum albumin reagent (BSA, Sigma Chemicals) as the standard.

### 2.6. Effects of pH and Temperature on Lipase Activity and Stability

To determine the effect of pH on lipase activity and stability, various pH buffer systems were used: citrate phosphate buffer (pH 5.0-6.0), Tris-HCl buffer (pH 7.0–9.0), and glycine-NaOH buffer (pH 10.0–10.6). To evaluate the pH stability, aliquots of enzyme samples were preincubated at 30°C for 1 h and 2 h with the respective pH buffers. Remaining enzyme activity was measured by using the standard* p*-NPP method and calculated while considering the initial activity as 100%.

The effect of temperature on lipase activity was studied by performing the enzyme reaction at different temperatures in the range of 10–70°C at pH 8.0. Similarly the thermal stability of enzyme was evaluated by measuring the residual activities at different time intervals (1-2 h) after incubating the enzyme solution at various temperatures (10–70°C). The residual activity was measured according to the p-NPP method as described earlier. The initial lipase activity was considered to be 100%.

### 2.7. Effects of Metal Ions and Enzyme Inhibitors on Lipase Activity

The effect of various metal ions (ZnCl_2_, CoCl_2_, NiCl_2_, NaCl, MnCl_2_, MgCl_2_, CaCl_2_, CdCl_2_, and CuCl_2_) and enzyme inhibitors (EDTA, *β*-mercaptoethanol, PMSF, iodoacetic acid, and SDS) on the lipase activity was investigated at the final concentration of 5 mM and 0.1%, respectively. The enzyme was preincubated with any of the selected chemicals for 1 h at 30°C. The residual lipase activity was measured against control (100%, no chemical added).

### 2.8. Organic Solvent Stability of Lipase

The effects of various organic solvents (e.g., methanol, ethanol, isopropanol, acetone, acetonitrile, dimethyl sulphoxide (DMSO), butanol,* n*-hexane,* n*-heptane, isooctane, chloroform, and ethyl acetate) on the enzyme were investigated. The lipase was incubated in the presence of (50% v/v) organic solvents at 30°C, 130 rpm for 1 h. The control was the sample without organic solvent under the same experimental conditions. Residual activity was measured using a standard method with* p*-NPP as the substrate. Residual activity was expressed by taking the activity without any organic solvent as 100%.

Besides this, the effects of various concentrations of methanol and ethanol and the incubation period of presence of these solvents were similarly measured.

### 2.9. Storage Stability of Lipase

The storage stability of the lipase was evaluated by measuring its activity (towards* p*-NPP) for 30 days at various time intervals of storage at 4°C.

### 2.10. Biodiesel Production Potential of Lipase

Biodiesel production was studied according to [11] with minor modifications [24]. Olive oil (7.89 mL) and methanol (0.99 mL) were kept in screw-capped glass tubes, mixed with a lipase sample (2.6 mL), and incubated at 40°C with shaking at 220 rpm for 48 h. After incubation, 200 µL of samples was taken from the reaction mixture and diluted with 1 mL of* n*-hexane for 2 min. Afterwards, the samples were centrifuged at 10 000 rpm for 15 min, and 10 µL of the upper layer was applied to a TLC plate. Methyl oleate (Sigma, purity 99%) was spotted as reference biodiesel. After developing the plate in* n*-hexane/ethyl acetate/acetic acid (90 : 10 : 1), the spots were visualized with iodine vapor after air drying for a short time.

## 3. Results

### 3.1. Screening of Lipolytic* Streptomyces* Isolates

In this study, the lipolytic activities of 300* Streptomyces* isolates, which were obtained from MUCC, were screened by using TA and Rhodamine-B Agar medium. In TA, all of the isolates were showed lipolytic activity. Although 270 isolates give a positive signal in Rhodamine-B Agar medium.

### 3.2. Quantitative Determination of Lipase Activity of the Isolates

The quantitative lipase activities of the isolates which have lipolytic activities were measured through a spectrophotometer using* p*-NPP as a substrate. After the screening of lipase activity* S. bambergiensis* OC 25-4 strain showed the highest lipase activity. The strain of* S. bambergiensis* OC 25-4 used in this study was selected among 300 strains of Streptomyces from MUCC as the best lipase producer.

### 3.3. Optimum Growth Conditions

The optimum inoculum density of spore solution of* S. bambergiensis* OC 25-4 was determined as 0.6 OD. The maximum lipase activity was observed when olive oil was used as a carbon and meat peptone was used as a nitrogen source. The optimum MgSO_4_ and CaCO_3_ rate of the media were determined as 0.1% and 0.01%, respectively (data not showed). The optimum time course of lipase production of* S. bambergiensis* OC 25-4 was determined and the optimum incubation time was found (120 h).

### 3.4. Enzyme Purification

The isolate was incubated until the optimum time and the culture supernatant were used in the purification stage. LipSB 25-4 was partially purified using a combination of ammonium sulfate precipitation, dialysis, and gel filtration column chromatography with Sephacryl S-100 HR. The purification results are summarized in Table 1. About 2.73-fold purification with 0.53% recovery was achieved. The specific activity of this enzyme was 92.12 U/mg.

### 3.5. Effects of pH and Temperature on Lipase Activity and Stability

**Figure 1:**
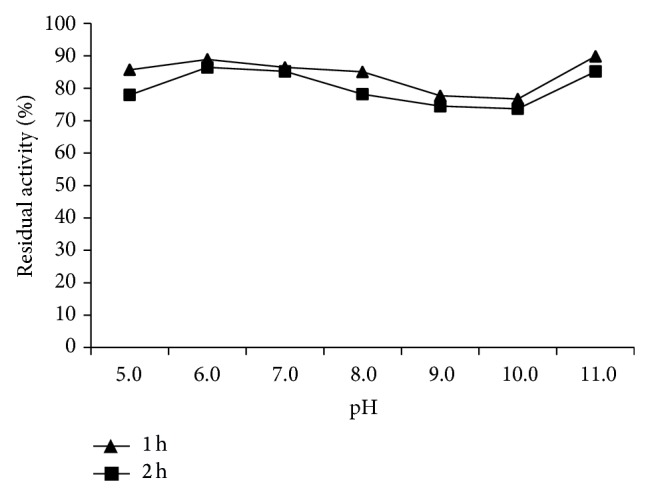
pH stability of LipSB 25-4.

**Figure 2:**
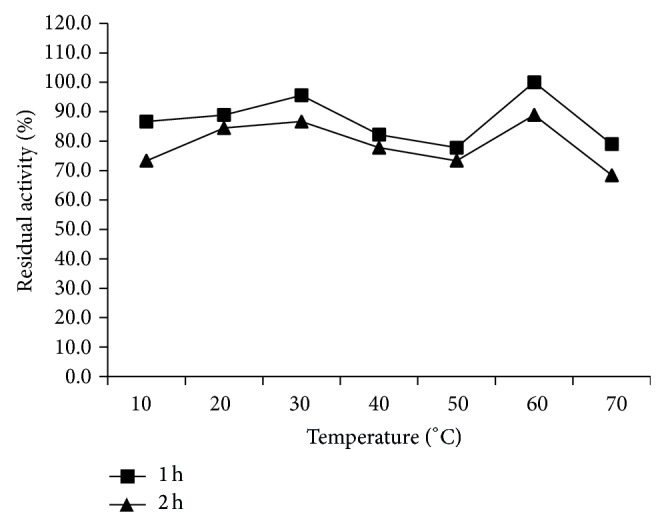
Temperature stability of LipSB 25-4.

The pH and temperature activity profile of the LipSB 25-4 was determined using different buffers of varying pH values and various temperatures. The enzyme exhibited maximum activity at pH 8.0 and 50°C. The enzyme activity was stable in pH ranges of 6.0-7.0. Almost 95% of the maximum activity remained stable at these pH values (Figure 1). The lipase was fairly active in between 10 and 70°C and the enzyme retained about 70% activity (Figure 2).

### 3.6. Effect of Metal Ions and Enzyme Inhibitors on Lipase Activity

The effects of various metal ions on LipSB 25-4 activity were tested (Table 2). The lipase was activated in the presence of Zn^2+^(114.3%) and completely inhibited in the presence of Mg^2+^. All the other tested metal ions suppressed the enzyme activity variously. The effects of enzyme inhibitors on LipSB 25-4 activity were tested. The results are shown in Table 2. While LipSB 25-4 was stable in the presence of EDTA and SDS, it was activated by *β*-mercaptoethanol and slightly inhibited by PMSF.

### 3.7. Organic Solvent Stability of Lipase

The effects of various organic solvents on LipSB 25-4 activity are presented in Table 2. The enzyme was generally highly stable in the presence of organic solvents. In the experiments carried out with organic solvents, it was seen that acetone (108.4%), acetonitrile (102.2%),* n*-heptane (107.0%), isooctane (114.4%), chloroform (101.9%), and ethyl acetate (108.4%) activated this enzyme.

**Figure 3:**
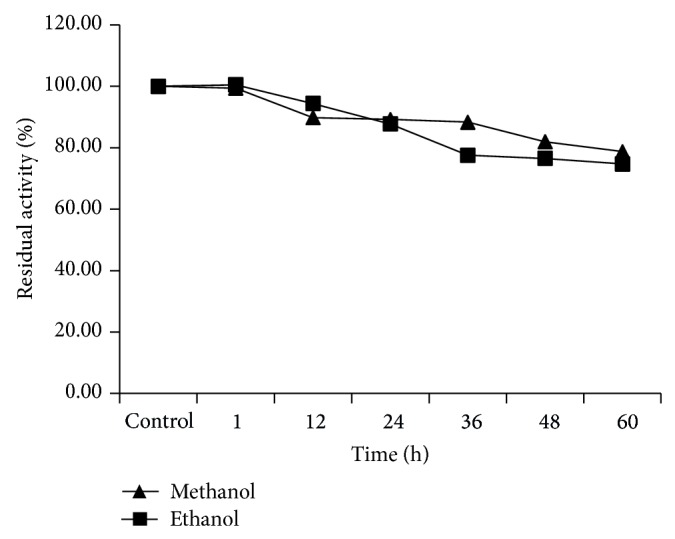
LipSB 25-4's stability at various time intervals in the presence of ethanol and methanol.

The effects of various concentrations of methanol and ethanol and the incubation period of presence of these solvents are presented in Figure 3. LipSB 25-4 was stable in the presence of methanol (over 80%) and ethanol (74%).

### 3.8. Storage Stability of Lipase

When LipSB 25-4's storage stability was examined, 73% of its initial activity after 30-day storage at 4°C was retained (data not shown).

### 3.9. Biodiesel Production Potential of LipSB 25-4

**Figure 4:**
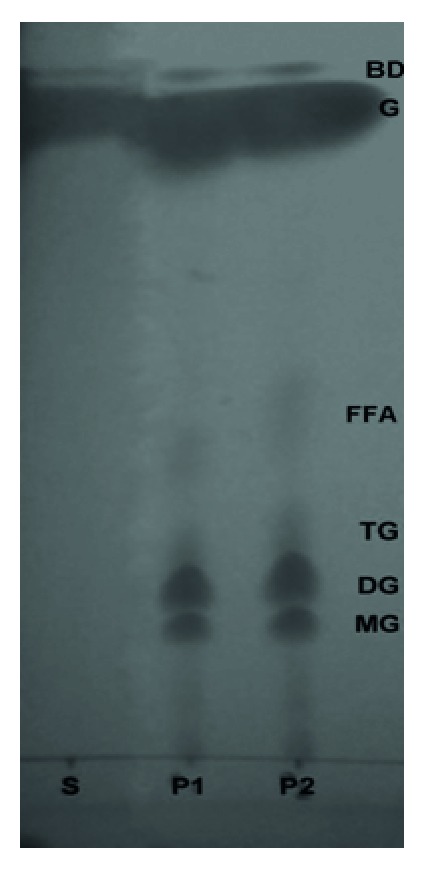
LipSB 25-4 catalyzed biodiesel production, TLC plate. S: standard (methyl oleate, Sigma Chemicals, 99% Pure), P1 and P2: reaction samples. BD: biodiesel, G: glycerol, FFA: free fatty acid, TG: triglyceride, DG: diglyceride, MG: monoglyceride.

LipSB 25-4 catalyzed biodiesel production in the presence of methanol and olive oil (Figure 4). The result indicates that LipSB 25-4 catalysed biodiesel production and it would be highly applicable in commercial production of biodiesel.

## 4. Discussion

During the past decades worldwide petroleum consumption has permanently increased due to the growth of human population and industrialization, which has caused depleting fossil fuel reserves and increasing petroleum price [25]. Biodiesel is an alternative fuel for diesel engines from renewable resources. Biocatalytic synthesis is considered a promising approach for biodiesel production with its key advantages over the conventional chemical catalyzed reactions.

In this study, characterization and potential use of alkaline and thermoactive lipase produced by* S. bambergiensis* OC 25-4 selected from 300* Streptomyces* isolates for biodiesel production were investigated. The lipase activity was observed at the beginning of incubation and reached the maximum value at 120 h. In another study,* Streptomyces caelestis* DSM 40084,* Streptomyces lavendulae* DSM 40708, and* Streptomyces lipmanii* DSM 40070 exhibited maximum lipase activity at 5 days [26].

LipSB 25-4 was partially purified with ammonium sulphate precipitation, dialysis, and gel filtration chromatography 2.73-fold and with 92.12 U/mg specific activity. The* S. bambergiensis* OC 25-4 strain was found to produce an alkaline thermoactive lipase with optimal activity at pH 8.0 and a temperature of 50°C. [27, 28] previously reported an extracellular lipase from* S. rimosus* and SCO7513 lipase from* S. coelicolor* A3(2) with optimal temperatures at 45°C and 45–55°C, respectively.

The lipase exhibited high stability in all tested values. Similarly, when* Streptomycetes* lipases were examined, pH stability has been found between [27] pH 4.0–10.0, [28] pH 6.0–11.0, and [29] pH 4.0–9.5. LipSB 25-4's temperature stability was determined and it was found that lipase is stable at 10–70°C. Temperature stabilities of* Streptomycetes* lipases are [27] fully thermostable [28], between 15 and 60°C [29], 55 and 65°C.

Among the cations tested, only the addition of Zn^2+^ resulted in increase in the lipase activity by 114.3. All the other tested metal ions suppressed the enzyme activity variously. In general, lipases were understood to be stimulated in the presence of Ca^2+^ and have been attributed to structural alterations rather than catalytic roles [30]. However, unlike such effect, the activity of LipSB 25-4 was reduced in the presence of Ca^2+^. Similar results obtained* S. fradiae* var. k11 and* Streptomyces* sp. CS133 lipase activities [31, 32]. The inhibitory nature of transition metals has been thought to be due to the interaction of ions with charged side-chain groups of surface amino acids, thus influencing the conformation and stability of the enzyme [33].

The enzyme was slightly reducted in the presence of PMSF and this result indicated the involvement of serine residues in the enzyme active site [27, 34], and this situation also observed the similar effect of PMSF on lipase from* Streptomyces rimosus*. Since the presence of EDTA did not abolish the activity it can be said that LipSB 25-4 is not a metalloprotein. Similarly some lipases are stable in presence of EDTA [24, 35]. Mercaptoethanol, however, stimulated lipase activity.

Stability in the presence of organic solvents is a requisite property of enzymes used in nonaqueous system. However, many enzymes are easily inactivated or denature in organic solvents and generally, enzymes are not stable in the presence of hydrophilic solvents [36]. However, our lipase showed significantly solvent-stable characteristics in both hydrophobic and hydrophilic solvent and almost unaffected in the presence of 50% organic solvents.

The transesterification reaction is the best method for production and modification of biodiesel. LipSB 25-4's characteristic properties and organic solvent stability show us that it is useful for transesterification reaction and biodiesel production as a biocatalyst. For this purpose enzyme stability in various concentrations of methanol and ethanol and enzyme stability at various time intervals were examined due to the fact that enzyme stability in these solvents is highly desirable for the enzymatic biodiesel production. Most importantly, lipase was adequately stable in methanol and ethanol retaining more than two-thirds of its activity for 60 h. Since methanol and ethanol are one of the popularly used organic media for biodiesel production, such stability is highly desirable.

The benefits of lipase catalyzed biodiesel synthesis include moderate reaction conditions, less alcohol required during the reaction, and less water and energy required for product isolation. LipSB 25-4 catalyzed transesterification reaction in the presence of methanol and olive oil. The requisite reaction time for various lipases is reported to be in the range 5–72 h [1].

## 5. Conclusion

As a result, in this study, LipSB 25-4 was partially purified from* S. bambergiensis* OC 25-4. Biochemical characterization of LipSB 25-4 showed that it has a number of industrially important characteristics The enzyme has high activity and stability at alkaline pH, and also stable in the presence of some metal ions and organic solvents. Its organic solvent tolerance capability was exploited to seek application in enzymatic biodiesel production. The lipase was found to catalyze biodiesel synthesis from olive oil. This is the first report of the purification, characterization, and potential application of* S. bambergiensis* lipase.

## Figures and Tables

**Table 1:** Summary of LipSB 25-4 purification.

Purification step	Total protein (mg)	Total activity (units)	Specific activity (U/mg)	Yield (%)	Fold purification
Crude extract	90.50	3057.47	33.78	100	1
Ammonium sulfate precipitation + dialysis	1.80	149.78	83.21	4.90	2.47
Gel filtration Chromatography	0.1764	16.25	92.12	0.53	2.73

**Table 2:** Effects of different metal ions, enzyme inhibitors, and organic solvents on LipSB 25-4 activity.

Reagent	Residual activity (%)
Control	100
Metal ions	
ZnCl_2_	114.3
CoCl_2_	74.6
NiCl_2_	76.2
NaCl	54.0
MnCl_2_	19.0
MgCl_2_	0.0
CaCl_2_	41.3
CdCl_2_	57.1
CuCl_2_	60.3
Enzyme inhibitors	
EDTA	94
*β*-Mercaptoethanol	108
PMSF	81
Iodoacetic acid	88
SDS	80
Control	100
Organic solvents	
Methanol	100.4
Ethanol	99.6
Isopropanol	91.1
Acetone	108.4
Acetonitrile	102.2
DMSO	96.9
Butanol	97.7
Hexane	99.5
*n*-Heptane	107.0
Isooctane	114.4
Chloroform	101.9
Ethyl acetate	108.4

Lipase preparation was incubated in the presence of 5 mM metal ions, 0.1% enzyme inhibitors, and 50% (v/v) organic solvents for 1 h at 30°C. Residual activity was measured using a standard method with *p*-NPP and the activity of enzyme without added metal ions, enzyme inhibitors, and organic solvent was taken as 100%.
